# New Biomaterials in Drug Delivery and Wound Care

**DOI:** 10.1155/2015/320496

**Published:** 2015-10-07

**Authors:** Šeila Selimović, Francesco Piraino, Robert Gauvin, Hojae Bae

**Affiliations:** ^1^American Association for the Advancement of Science, Arlington, VA 22202, USA; ^2^Institute of Bioengineering, School of Engineering, École Polytechnique Fédérale de Lausanne, 1015 Lausanne, Switzerland; ^3^Quebec Center for Functional Materials and Department of Surgery, Faculty of Medicine, Université Laval, Quebec City, QC, Canada G1V 0A6; ^4^Department of Bioindustrial Technologies, College of Animal Bioscience and Technology, Konkuk University, Seoul 05029, Republic of Korea

Biological organisms evolve within specific conditions and constraints in their environment, giving rise to elegant and efficient strategies for fabricating materials that often outperform man-made materials of similar composition. Synthesizing and engineering novel bioinspired materials with similar characteristics require in-depth understanding of the properties, composition, and hierarchical organization of biological materials. These biomaterials are becoming increasingly incorporated into the medical sector, for example, as transport vehicles in drug delivery or as skin and soft tissue substitutes in wound management.

In this view, several investigators have been invited to contribute original research findings and reviews that could stimulate continuing efforts to understand new biomaterials with direct clinical applications in the interconnected fields of wound care, drug encapsulation, and transport. This special issue is divided into categories based on the following key words: stem cells, bone tissue engineering, functional biomaterials, scaffolds, and implants.

In the category of stem cells, J.-M. Bourget et al. evaluated the potential use of mesenchymal stem cells (MSCs) isolated from bone marrow-derived MSCs and umbilical cord blood-derived MSCs for blood vessel engineering. Their results reinforce the versatility of the self-assembly approach since they demonstrate that it is possible to recapitulate a contractile media layer from MSCs, without the need of exogenous scaffolding material. E. A. Wahl et al. compared the behavior of human adipose-derived MSCs seeded on four different biomaterials that are awaiting or have already received Food and Drug Administration approval to determine a suitable regenerative scaffold for delivering these cells to dermal wounds and increasing wound healing potential.

In the category of bone tissue engineering, I. Ortega-Oller et al. reported the use of nano- and/or microparticles of poly(lactic-co-glycolic acid) (PLGA) as a delivery system of the bone morphogenetic protein 2. R. Ramos-Zúñiga et al. concentrated on the intrinsic properties offered by chitosan and its use in tissue engineering, considering it as a promising alternative for regenerative medicine as a bioactive polymer. M. K. Wasko and R. Kaminski performed a systematic review of current evidence of antibiotic cement nails (ACNs) in orthopedic trauma and provided an up-to-date analysis of the indications, operative technique, failure mechanisms, complications, outcomes, and outlooks for the ACNs use in long bone infection.

In the category of functional biomaterials, F. Piraino and Š. Selimović discussed approaches in tissue engineering and regenerative medicine to address stages characterizing the mammalian response to tissue injury through the application of biomaterials. They also reviewed molecular therapies with particular attention to drug delivery methods and gene therapies. Finally, they examined cellular treatments and provided an outlook on the future of drug delivery and wound care biomaterials. I. Fukuda et al. synthesized two types of mannose-modified lipids with different stereoisomer (*α*-mannose and *β*-mannose). Their delivery system to macrophages may overcome the problems for gene therapy and could be potentially used for treatment of immune diseases involved in macrophages.

In the category of scaffolds, A. M. Eweida and M. K. Marei reviewed the online published literature for the studies that performed extracellular matrix (ECM) revitalization believing that, in chronic and difficult-to-heal wounds, revitalizing the ECM scaffolds would be beneficial to overcome the defective host tissue interaction. H. Akagi et al. evaluated the utility of a hydroxyapatite (HA) and poly-DL-lactide (PDLLA) scaffold compared to *β*-tricalcium phosphate, at a loading site as a new bioresorbable scaffold.

Finally in the category of implants, S. Abdolrahimzadeh et al. evaluated the effectiveness of one or two intravitreal injections of a sustained release dexamethasone implant in patients with persistent macular edema following uncomplicated phacoemulsification. They observed a statistically significant improvement of mean central foveal thickness and best corrected visual acuity with one or two intravitreal dexamethasone implants over 12 months.

By collecting these papers, we hope to enrich our readers and researchers in the field of biomaterials in drug delivery and wound care. We believe that new biomaterials will be an important part of future drug delivery and wound care research activities.

